# Effect of Korean Red Ginseng on Cholesterol Metabolites in Postmenopausal Women with Hypercholesterolemia: A Pilot Randomized Controlled Trial

**DOI:** 10.3390/nu12113423

**Published:** 2020-11-08

**Authors:** Yu-Jin Kwon, Su-Nyeong Jang, Kwang-Hyeon Liu, Dong-Hyuk Jung

**Affiliations:** 1 Department of Family Medicine, Yongin Severance Hospital, Yonsei University College of Medicine, Yongin 16995, Korea; digda3@yuhs.ac; 2 BK21 Plus KNU Multi-Omics based Creative Drug Research Team, College of Pharmacy and Research Institute of Pharmaceutical Sciences, Kyungpook National University, Daegu 41566, Korea; wts1424@naver.com; 3 College of Pharmacy and Research Institute of Pharmaceutical Sciences, Kyungpook National University, Daegu 41566, Korea; dstlkh@gmail.com

**Keywords:** Korean red ginseng, cholesterol metabolism, sterols, menopause, women

## Abstract

Korean red ginseng (KRG) is known to exert beneficial effects on cardiovascular health. Meanwhile, reduced estrogen at menopause has been shown to have various adverse impacts on cardiovascular risk factors, including blood lipids. The aim of this pilot study was to investigate the effect of KRG on cholesterol metabolites, which are surrogate markers of cholesterol absorption and biosynthesis, in postmenopausal women with hypercholesterolemia. The present study is an exploratory study which used data from a 4-week, double-blinded, placebo-controlled clinical pilot study in 68 postmenopausal women with hypercholesterolemia. Patients received KRG (2 g) or placebo (2 g) once daily. The primary endpoints were changes in the levels of nine sterols. Serum sterols were analyzed using liquid chromatography-mass spectrometry (LC-MS)/MS analysis. Among the sterols, reduction in cholesterol level were significantly larger in the KRG group than in the placebo group (the changes: −148.3 ± 261.1 nmol/mL in the ginseng group vs. −23.0 ± 220.5 nmol/mL in the placebo group, *p* = 0.039). Additionally, changes in 7-hydroxycholesterol (7-OHC) were significantly larger in the KRG group than in the placebo group (the changes: −0.05 ± 0.09 nmol/mL in the ginseng group vs. −0.002 ± 0.1 nmol/mL in the placebo group, *p* = 0.047). Oxysterols, cholesterol derivates, have been known to play a role in chronic inflammation diseases such as cardiovascular diseases. KRG improves sterol metabolism by decreasing cholesterol and 7-OHC levels in postmenopausal women with hypercholesterolemia.

## 1. Introduction

Cardiovascular disease (CVD), particularly ischemic heart disease, remains the global leading cause of death in older women [1]. During the aging process, changes in blood lipids contribute to an increased risk of developing CVD. After 20 years of age, total blood cholesterol levels increase continuously in men and women. In men >50 years of age, blood cholesterol levels plateau, whereas in women >40 years of age, blood cholesterol levels increase progressively [2]. In women between 40 and 60 years of age, low-density lipoprotein (LDL) cholesterol levels increase ~0.05 mmol/L per year; thus, between the ages of 55–60 years, LDL cholesterol levels are greater in women than in men [2,3]. Meanwhile, menopause, which is a risk factor for CVD and associated with low estrogen levels, can have various negative effects on cardiovascular function and metabolism [4]. Estrogen regulated liver lipid metabolism by reducing de novo lipogenesis [5].

Korean red ginseng (KRG) is derived from the root of the *Panax ginseng* Meyer plant, which is widely used in not only Asian countries but also North American and Europe as a functional health food [6,7]. Many studies have shown the beneficial effects of KRG on fatigue and blood circulation, including anti-oxidant, anti-inflammatory, and anti-cancer effects [7,8,9]. Several studies have shown that KRG has beneficial effects on CVD [10]. Although two clinical trials have evaluated the effects of KRG on lipid profiles in postmenopausal women [11,12], studies evaluating the effects of KRG on human lipid profiles have yielded inconsistent results [11,12,13,14], and a few studies have focused on cholesterol metabolism. Our study included the postmenopausal women with hypercholesterolemia and analyzed cholesterol metabolites using the liquid chromatography–mass spectrometry (LC-MS)/MS analysis.

Cholesterol is found in many biological membranes and is the main animal sterol [15]. Cholesterol metabolites are important molecules that regulate metabolism, development, and homeostasis in humans [16]. Dysfunction in metabolism of cholesterol, sterol intermediates, and oxysterols occurs unfavorable effects in atherosclerosis [17]. Macrophage cholesterol accumulation leads to foam cell formation and inflammation, contributing to CVD [17]. Oxysterols are also closely associated with atherosclerosis contributing pathway [18]. Therefore, we may be able to determine the underlying mechanisms for the effects of KRG on hypercholesterolemia by understanding sterols. The aim of this pilot study was to investigate the effect of KRG on serum sterols in postmenopausal women with hypercholesterolemia.

## 2. Materials and Methods

### 2.1. Study Design and Patients

The present study is an exploratory study which used data from following original study (Clinical Research Information Service [CRIS], KCT0003927). The design of original study was a 4-week, randomized, double-blind, placebo-controlled, parallel-arm pilot clinical trial. The cholesterol metabolites were analyzed only in the participants who completed this study. The protocol was approved by the Institutional Review Board of Yongin Severance Hospital (IRB No. 9-2018-0011). This study was performed in compliance with the Declaration of Helsinki. Written consent was obtained from all patients prior to participation. Patients were recruited from October 2018 to May 2019 at Yongin Severance Hospital (Yongin, Korea). We recruited the participants who visited the Yongin Severance health check-up center and family medicine clinic. Postmenopausal women who were reported to have high cholesterol level (serum total cholesterol ≥200 mg/dL or serum LDL cholesterol ≥130 mg/dL) within 1 month were recruited in this study. At the screening visit, participants underwent blood test and those who have with serum total cholesterol ≥200 mg/dL or serum LDL cholesterol ≥130 mg/dL were enrolled in this study. Menopause was defined as 12 consecutive months of no menstrual period based on the World Health Organization definition [19]. Participants were excluded if they had CVD, cerebrovascular disease, progressive atherosclerosis, uncontrolled hypertension (BP > 180/120 mmHg) [20,21] uncontrolled diabetes (fasting glucose of 11.1 mmol/L), hepatic disease, or renal disease, if they were taking lipid lowering drugs, if they were taking hormone replacement therapy, or if they were allergic to ginseng. Medical history, drug history and menopause status were self-reported. The presence of hypertension was determined as a blood pressure of systolic ≥140 or diastolic ≥90 mm Hg, or taking anti-hypertensive medication. The presence of diabetes was determined as fasting glucose 126 mg/dL or above, or taking anti-diabetic medication.

### 2.2. Randomization and Masking

**Figure 1:**
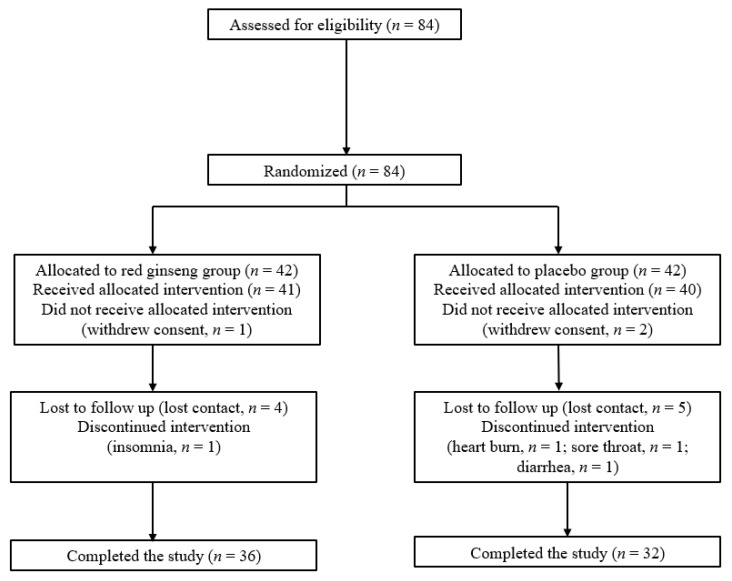
Flow chart of the study population.

Participants were assigned randomly (in a 1:1 ratio) to receive four KRG tablets (2 g) or four placebo tablets (2 g) each day. Randomization was performed using a centralized computer-generated system. Investigators and patients were masked to the assigned treatment throughout the study. KRG and placebo tablets were identical in appearance and produced by the study sponsor (Korea Ginseng Corporation, Seoul, Korea). Each KRG tablet contained 500 mg of KRG, which contains numerous major ginsenosides (Rg1: 2.89%, Re: 2.16%, Rf: 0.93%, Rh1: 0.13%, Rg2: 0.58%, Rb1: 5.16%, Rc: 2.22%, Rb2: 1.82%, Rd: 0.47%, Rg3: 0.22%). The placebo group was provided with identically shaped tablets containing over 90% corn starch. Of the initial 84 participants assessed for eligibility and enrolled in the study, 42 participants were assigned to receive KRG, and 42 participants were assigned to receive the placebo. The flow chart was described in Figure 1. The adverse event reported in the KRG group was insomnia (*n* = 1), and the adverse events reported in the placebo group were heartburn (*n* = 1), sore throat (*n* = 1), and diarrhea (*n* = 1). The study was completed by 36 participants in the KRG group and 32 participants in the placebo group.

### 2.3. Procedures and Endpoints

Participants underwent three examinations: initial screening, at baseline, and at 4 weeks. Body weight, waist circumference (WC), systolic blood pressure (SBP), and diastolic blood pressure (DBP) were measured at each visit. Height and weight were obtained with participants wearing light indoor clothing and no shoes to the nearest 0.1 cm (Seca 225, Hamburg, Germany) and 0.1 kg, respectively (GL-6000-20, G-tech, Gyeonggi, Korea). Waist circumference was measured at the midway between the costal margin and the iliac crest at the end stage of a normal expiration (Seca 200, Hamburg, Germany). Systolic and diastolic blood pressure were measured three times in a seated position after a 5-min rest time using a Heine Gamma^®^ G7 aneroid sphygmomanometer (Heine Optotechnik, Herrsching, Germany). Cholesterol metabolites were measured at baseline and at 4 weeks. Physical activity, smoking, and alcohol consumption questionnaires were administered at baseline and at 4 weeks. Participants were categorized into non-smoker and current smoker groups. An alcohol drinker was defined as a person who drinks alcohol more than once a month. Physical activity was defined as performing light to moderate exercise more than two times per week. We used binary variables of presence or absence for the history of hypertension and diabetes on a self-reported questionnaire. Body mass index (BMI) was calculated by dividing weight (kg) by height (m^2^). Blood samples were obtained after >8 h of fasting. At each visit, all participants were told to maintain their usual lifestyle. The medical staff telephoned the participants every week to check for adverse effects and compliance. At the last visit, information was obtained on the number of pills returned. The difference between the number of pills received at the first visit and the number of pills returned represented compliance. The primary efficacy endpoints were changes in nine sterols (cholesterol, sitosterol, campesterol, lanosterol, desmosterol, 7-dehydrocholesterol, 7-hydroxycholesterol, CE 14:0, CE 20:4). Safety assessment consisted of an assessment of adverse events, measurement of vital signs, including SBP, DBP, and heart rate (HR), and laboratory tests for aspartate aminotransferase and alanine aminotransferase. The compliance rate was >80%.

### 2.4. Reagents

The cholesterol-d7 (>99%) reference standards were purchased from Avanti Polar Lipids (Alabaster, AL, USA). Desmosterol (>84%), 7-dehydrocholesterol (7-DHC, >95%), 7-hydroxycholesterol (7-OHC, >95%), cholesterol (>99%), lanosterol (>93%), stigmasterol (95%), campesterol (65%), ammonium acetate, butylated hydroxytoluene (BHT), and methyl-tert-butyl ether (MTBE) were obtained from Sigma-Aldrich (St. Louis, MO, USA). Cholesteryl ester 20:4 (>98%) and cholesteryl ester 14:0 (>98%) were purchased from Cayman Chemical Company (Ann Arbor, MI, USA). Water, methanol, chloroform, and isopropanol were liquid chromatography–mass spectrometry (LC-MS) grade (Fisher Scientific Co., Pittsburgh, PA, USA).

### 2.5. Lipid Extraction and LC-MS/MS Analysis

Sterols and cholesteryl esters from human plasma were extracted using the Matyash method with some modifications [22]. The tube used for lipid extraction was Eppendorf tube (Eppendorf safe-lock tube 2.0 mL, product number: 0030 120 094). Briefly, 100 μL of human plasma was added to an Eppendorf tube containing cholesterol-d7 (internal standard, 500 ng), followed by the addition of 400 μL of ice-cold 75% methanol with 0.1% BHT and 1 mL MTBE, and shaken for 1 h at room temperature with 2000 rpm speed (using the Multi Reax shakers and mixers). For phase separation, 250 μL of water was added, and the sample was centrifuged at 14,000 g at 4 °C for 15 min. The upper phase was transferred to a new tube and dried under vacuum. Lastly, the sample was reconstituted in 100 μL of chloroform/methanol (1:9, *v*/*v*). Quantitative analysis of sterols was performed using a Nexera2 LC system (Shimadzu Corporation, Kyoto, Japan). The LC system was connected to a triple quadrupole mass spectrometer (LC-MS 8060; Shimadzu, Kyoto, Japan) with a reversed phase Kinetex C18 column (100 × 2.1 mm, 2.6 μm, Phenomenex, Torrance, CA, USA) for chromatographic separation of sterols. Mobile phase A consisted of water/methanol (1:9, *v*/*v*) containing 10 mM ammonium acetate, and mobile phase B consisted of isopropanol/methanol (5:5, *v*/*v*) containing 10 mM ammonium acetate. The gradient elution program was as follows: 0 min with 30% B, 0–15 min with 95% B, 15–20 min with 95% B, and 20–25 min with 30% B. The total run time was 25 min, and the flow rate was 0.2 mL/min. For each run, 5 μL of sample was injected. An electrospray ionization interface was used as the ionization source in the positive mode. The optimum operating conditions were as follows: capillary temperature, 350 °C; vaporizer temperature, 300 °C; collision gas (argon) pressure, 1.5 mTorr. To determine the concentration of each target sterol, the calculated ratio of target analyte and internal standard (IS) was multiplied by the concentration of the IS [23,24]. The selective reaction monitoring transitions and collision energies determined for each sterol are listed in Table S1. The concentration of stigmasterol was not determined in this study.

### 2.6. Statistical Analysis

We analyzed the data based on the law of large number and central limit theorem. Data are presented as means ± standard deviations. We used data from the per-protocol set, which included participants who completed the trial. Differences in baseline characteristics between the KRG and placebo groups were compared using independent *t*-tests. Differences after intervention within groups were compared using paired t-tests. Differences between the two groups were compared using independent t-tests. Differences of sterols between the two group were also compared using the analysis of covariance after adjusting each baseline value of sterols. Significance tests were two-sided with an alpha value of 0.05. All statistical analyses were performed using SPSS software version 25.0 (IBM Corp., Armonk, NY, USA).

## 3. Results

A total of 68 participants completed this study. The study dropout rates were similar between the two groups. The baseline characteristics of the two groups are described in Table 1. The baseline characteristics were similar between the KRG (*n* = 36) and placebo (*n* = 32) groups in regards to age (55.9 ± 5.9 years vs. 58.1 ± 4.7 years, respectively, *p* = 0.093), BMI (24.3 ± 3.2 kg/m^2^ vs. 24.5 ± 3.7 kg/m^2^, respectively, *p* = 0.741), and WC (82.5 ± 8.7 cm vs. 82.6 ± 10.2 cm, respectively, *p* = 0.950). There were also no differences in SBP, DBP, heart rate, white blood cell, aspartate aminotransferase, and alanine aminotransferase between the two groups. The proportion of participants with hypertension or diabetes were also similar between the KRG and placebo groups (hypertension, 13.9% vs. 15.6%, respectively, *p* = 0.572; diabetes, 5.6% vs. 3.1%, respectively, *p* = 0.535). There were also no significant differences in physical activity, smoking, or alcohol consumption between the two groups. The baseline levels of the nine sterols tested did not differ significantly between the two groups.

The changes in the metabolic parameters in the two groups before and after 4 weeks of intervention are shown in Table 2. KRG group showed a decreasing trend in SBP (119.8 ±13.5 mmHg vs. 116.6 ± 15.1 mmHg, *p* = 0.056). DBP was significantly decreased in the KRG group (76.7 ± 9.7 mmHg vs. 73.1 ± 8.8 mmHg, *p* = 0.017). However, the mean changes in SBP and DBP between the KRG and placebo groups were not different. BMI and WC did not change significantly.

Table 3 shows the changes in sterols in the two groups before and after intervention. Among the sterols, cholesterol levels were significantly lower in the KRG group (1634.4 ± 409.4 nmol/mL vs. 1486.1 ± 312.8 nmol/mL, *p* = 0.002), but not in the placebo group (1510.6 ± 339.2 nmol/mL vs. 1486.6 ± 388.3 nmol/mL, *p* = 0.543). The mean change in cholesterol levels was significantly greater in the KRG group than the placebo group (KRG: a change of −148.3 ± 261.1, placebo: a change of −23.0 ± 220.5, *p* = 0.039). This significant result remained after adjusting for baseline value of cholesterol level (*p* = 0.047). Changes in 7-OHC levels in the KRG group was significant (0.3 ± 0.2 vs. 0.2 ± 0.2, *p* = 0.002), whereas the placebo group showed no change (0.2 ± 0.6 vs. 0.2 ± 1.4, *p* = 0.908). The mean change in 7-OHC levels was significantly greater in the KRG group, compared with the placebo group (KRG: a change of −0.05 ± 0.09, placebo: a change of −0.002 ± 0.1, *p* = 0.047). The significance was attenuated after adjusting for baseline value of 7-OHC (*p* = 0.063). There were no significant differences in other sterols, including sitosterol, campesterol, lanosterol, desmosterol, 7-DHC, CE14:0, and CE20:4, after four weeks of intervention

## 4. Discussion

We found that intervention with KRG led to favorable changes in cholesterol and 7-OHC levels in postmenopausal women with hypercholesterolemia. This is an exploratory study from the original clinical study. In the primary analysis, the mean changes in traditional total cholesterol were −16.0 ± 27.9 mg/dl in KRG group and −5.9 ± 17.1 mg/dl in placebo group, respectively (*p* = 0.067). The mean changes in the low-density lipoprotein cholesterol were −11.8 ± 20.6 mg/dl in KRG group and −3.3 ± 14.6 in placebo group, respectively (*p* = 0.054) (data not shown). Quantification of traditional lipid profiles could be relatively imprecise due to cross reacting antibodies in immunoassays and interfering analytical signals in colorimetric/fluorometric detection systems [17]. Liquid chromatography-mass spectrometry (LC-MS)/MS analysis could help to quantify the cholesterol metabolites profiles [17,25]. Intracellular cholesterol homeostasis is regulated by various cholesterol precursor sterols [25]. Cholesterol intermediates and metabolites have also diverse role in pathophysiology of cardiovascular diseases [17]. Endogenous cholesterol synthesis is regulated by the mevalonate pathway via lanosterol [26]. Lanosterol is converted to cholesterol through two distinct pathways: the lathosterol and 7-DHC (Kandutsch-Russel) pathway and the desmosterol (Bloch) pathway [26]. Cholesterol is then metabolized to cholesterol esters and hydroxycholesterols. Sterols in plants commonly exist as a mixture of β-sitosterol, campesterol, and stigmasterol. 

In this study, changes in cholesterol and 7-OHC levels showed significant reductions in the KRG group when compared to the placebo group after 4 weeks of intervention (*p* = 0.039 and *p* = 0.047, respectively). Some animal studies support our results. A study in hyperlipidemic rabbits showed that ginseng saponins improve lipid profiles by increasing lipoprotein lipase activity [27]. In a rat study, black ginseng ameliorated hypercholesterolemia by attenuating mRNA levels of acetyl-coenzyme A (CoA) acetyltransferase 2, sterol regulatory element-binding protein 2, and 3-hydroxy-3-methyl-glutaryl-CoA reductase [28]. Some clinical studies in postmenopausal women are also in line with our results. Kim et al. [11] showed that total cholesterol and LDL cholesterol decreased significantly in postmenopausal women who took KRG (3 g/day) for 12 weeks (*n* = 72, 2 groups). Lee et al. [12] also showed that LDL cholesterol levels decreased significantly in the KRG group (2.1 g/day) for 2 weeks (*n* = 117, 2 groups). Several clinical studies did not find significant effect of RG on lipid metabolism. In the double-blinded clinical trial including 40 healthy participants, there were no statistically significant changes in lipid profiles in KRG group (1.5 g/day for 8 weeks) [29]. In a 12-week randomized, double-blinded, placebo-controlled (5 g of KRG [*n* = 21] or placebo [*n* = 20] in tablet form) trial in patients with impaired fasting glucose or diabetes, there were no significant lipid profile changes in the either group [14]. The difference in the KRG dose, study duration, study population could lead to inconsistent findings. 

Ginsenoside Rh1, an important component of KRG, has been shown to have estrogenic activity [30]. Cho et al. [31] showed that ginsenoside-Rb1 activated both estrogen receptor alpha (ERα) and estrogen receptor beta (ERβ), leading to transactivation of estrogen-responsive genes. Previous studies have indicated that oral administration of estrogen reduced LDL cholesterol levels by accelerating the conversion of hepatic cholesterol in bile acids and increasing the cellular expression of LDL receptors [32]. Other major ginsenosides Rb1, Rg1, and Rg3 have intrinsic property of controlling reactive oxygen species (ROS), nitric oxide (NO) production, and the ability to activate various receptors in endothelial cells [33]. In the vitro study, Rg3 reduces lipid accumulation with AMP-Activated Protein Kinase (AMPK) activation in HepG2 cells [34].

These studies support our results, in that KRG intervention reduced cholesterol levels in postmenopausal women.

Oxysterols, oxygenated derivatives of cholesterol, are intermediate and end products in cholesterol excretion pathways [35]. Oxysterols are known to be physiological mediators related to a number of cholesterol-induced metabolic effects [36]. The classic bile acid synthetic pathway starts with 7α-hydroxycholesterol, which is synthesized by the rate-limiting hepatic cytochrome *p*-450 enzyme, cholesterol 7α-hydroxylase (CYP7A1) [37]. The most abundant oxysterols generated through autoxidation are 7-ketocholesterol and 7β-hydroxy cholesterol, which are modified at the 7-position of the cholesterol B-ring [38]. There oxysterols have cytotoxic and pro-apoptotic properties [38]. Several oxysterols are found in macrophages and macrophage-derived ‘foam cells’ in atherosclerotic tissue and are involved in endothelium dysfunction, lipid accumulation, and arterial stiffness due to their marked pro-oxidant, pro-inflammatory, and pro-apoptotic properties [38]. Several experimental studies could support our study. Song et al. [39] showed that KRG modulates the expression of genes related to lipid metabolism, including oxysterol binding protein like-3. In an in vitro study, KRG was found to induce suppression of sterol regulatory element binding protein 1c (SREBP1c) and activation of peroxisome proliferator-activated receptor α (PPARα) via AMP-activated protein kinase (AMPK) in HepG2 cells [40]. Several studies have also found that KRG has biological functions, ameliorating various disease symptoms via antioxidant mechanisms in cells and animals [41]. The antioxidant effect of KRG could lead to favorable changes in oxysterols. Reduction of 7-hydroxycholesterol could prevent pathogenesis of vascular ageing through decreasing their marked pro-oxidant, proinflammatory and proapoptotic properties [37].

Our study has several limitations. First, our study was only conducted for 4 weeks, which is a relatively short period. Previous clinical studies associated with KRG involved various treatment durations from 2 weeks to 12 weeks. We previously have shown that KRG has a favorable effect on mitochondrial function and hormones in men with metabolic syndrome with 4 weeks of intervention [42]. Our colleagues thought that a 4-week intervention period might be short to observe a sufficient effect of KRG on sterols; however, there could be an advantage to recognizing the effect of KRG by helping control external factors. The optimal duration and schedule for RG intervention should be investigated in the further investigation. Second, although we told the participants to maintain their usual eating habits during the clinical trial, we could not control for diet, which could affect dietary cholesterol and plant sterol levels. Third, 7β-hydroxy cholesterol and 7α-hydroxy cholesterol were not separated in this study; therefore, we analyzed them as 7-OHC. Accurately identifying specific oxysterols is difficult due to an abundance of cholesterol and the possibility of artifactual formation of several oxysterols during isolation. Fourth, sterols are only one of eight classes of lipids. Further analyses that include all of the lipid classes are needed to understand lipid metabolism in relation to ginseng. Fifth, we did not conduct Bonferroni correction to interpret the results in an exploratory manner. In this exploratory study, we found the two meaningful sterol metabolites (cholesterol and 7-Hydroxycholesterol). The power was 0.557 (in case of cholesterol). Based on this pilot study, a corroborative randomized controlled trial with a sufficient sample size is needed. Sixth, the sterol metabolites were assessed in the participants who completed the original trial. Therefore there could be a selection bias. Finally, the results of this study can only be applied to postmenopausal Korean women and not to men, premenopausal women, or individuals of other races. Despite these limitations, this is the first double-blinded randomized controlled pilot trial to investigate the effect of KRG on cholesterol metabolites in postmenopausal women with hypercholesterolemia.

## 5. Conclusions

In conclusion, by measuring serum cholesterol metabolites, we found that KRG could have a favorable effect on cholesterol homeostasis in postmenopausal women with hypercholesterolemia. These findings suggest that KRG intake could improve cholesterol metabolism and prevent CVD in postmenopausal women with hypercholesterolemia. Further, well-designed, corroborative studies are needed to support our findings.

## Figures and Tables

**Table 1:** Baseline characteristics of the study participants.

	Ginseng	Placebo	*p*-Value
*n*	36	32	
Age, years	55.9 ± 5.9	58.1 ± 4.7	0.093
Physical measurement			
Body mass index, kg/m^2^	24.3 ± 3.2	24.5 ± 3.7	0.741
Waist circumference, cm	82.5 ± 8.7	82.6 ± 10.2	0.950
SBP (mmHg)	119.8 ± 13.5	116.8 ± 16.5	0.409
DBP (mmHg)	76.7 ± 9.7	72.2 ± 9.3	0.065
Heart rate (bpm)	76.8 ± 9.6	75.4 ± 11.4	0.585
WBC(×10^3^ L)	5.7 ± 1.4	5.8 ± 1.6	0.786
AST (IU/L)	24.5 ± 7.0	25.2 ± 6.0	0.654
ALT (IU/L)	21.7 ± 12.6	20.5 ± 7.8	0.629
Comorbid condition, *n* (%)			
Hypertension	5 (13.9)	5 (15.6)	0.572
Diabetes	2 (5.6)	1 (3.1)	0.535
Physical activity, *n* (%)	15 (41.7)	10 (31.3)	0.374
Smoking, *n* (%)	2 (5.6)	1 (3.1)	0.534
Alcohol consumption, *n* (%)	9 (25.0)	10(13.2)	0.567
Sterols			
Cholesterol (nmol/mL)	1634.5 ± 409.3	1510.6 ± 339.2	0.182
Plant sterols (nmol/mL)			
Sitosterol	8.2 ± 8.0	6.0 ± 3.2	0.159
Campesterol	11.3 ± 12.6	8.5 ± 4.8	0.245
Cholesterol precursor (nmol/mL)			
Lanosterol	2.9 ± 1.2	3.0 ± 1.2	0.626
Desmosterol	9.6 ± 2.4	10.7 ± 13.8	0.622
7-Dehydrocholesterol	6.2 ± 1.7	9.4 ± 22.2	0.392
Oxysterols (nmol/mL)			
7-Hydroxycholesterol	0.3 ± 0.2	0.2 ± 0.2	0.123
Cholesterol esters (nmol/mL)			
CE 14:0	14.6 ± 15.0	16.9 ± 19.0	0.572
CE 20:4	111.9 ± 124.8	99.1 ± 112.3	0.659

SBP, systolic blood pressure; DBP, diastolic blood pressure; WBC, white blood cell; AST, aspartate aminotransferase; ALT, alanine aminotransferase; CE, cholesterol esters. *p*-values were calculated using the independent two-sample *t*-test for continuous values and the chi-square test for categorical value.

**Table 2:** Changes in metabolic parameters and cholesterol metabolites in the ginseng and placebo groups before and after the 4-week intervention.

	Ginseng				Placebo				
Metabolic Parameters	Baseline	After 4 Weeks	p †	Change	Baseline	After 4 Weeks	*p* †	Change	*p* ‡
BMI (kg/m^2^)	24.3 ± 3.2	24.4 ± 3.0	0.315	0.1 ± 0.8	24.5 ± 3.7	24.5 ± 4.0	0.986	−0.00 ± 0.8	0.473
WC (cm)	82.5 ± 8.7	83.3 ± 8.8	0.145	0.9 ± 3.4	82.6 ± 10.2	83.1 ± 10.2	0.494	0.5 ± 3.7	0.648
SBP (mmHg)	119.8 ± 13.5	116.6 ± 15.1	0.056	−3.2 ± 9.7	116.8 ± 16.5	116.8 ± 17.5	0.989	0.03 ± 12.6	0.239
DBP (mmHg)	76.7 ± 9.7	73.1 ± 8.8	0.017	−3.6 ± 8.7	72.4 ± 9.3	71.6 ± 11.4	0.661	−0.8 ± 10.0	0.212
HR (bpm)	76.8 ± 9.6	76.1 ± 11.2	0.555	−0.3 ± 1.2	75.4 ± 11.4	74.7 ± 12.5	0.771	−0.7 ± 7.0	0.988
WBC $(\times 10^{3}$ L)	5.7 ± 1.4	5.5 ± 1.4	0.828	−0.05 ± 1.3	5.8 ± 1.6	5.5 ± 1.2	0.239	−0.3 ± 1.2	0.487
AST (IU/L)	24.5 ± 7.0	25.9 ± 18.2	0.659	1.4 ± 19.5	25.2 ± 6.0	25.9 ± 7.9	0.602	0.7 ± 7.7	0.844
ALT(IU/L)	21.7 ± 12.6	21.8 ± 21.8	0.989	0.1 ± 23.9	20.5 ± 7.8	19.8 ± 10.5	0.720	−0.7 ± 10.7	0.872

BMI, Body mass index; WC, Waist circumference; SBP, Systolic blood pressure; DBP, Diastolic blood pressure; HR, Heart rate; WBC, White blood cell; AST, Aspartate aminotransferase; ALT, Alanine aminotransferase.† *p*-values were calculated using the paired t-test (difference after intervention within groups), ‡ *p*-values were calculated using the independent two-sample *t*-test (mean change differences between two groups).

**Table 3:** Changes in metabolic parameters and cholesterol metabolites in the ginseng and placebo groups before and after the 4-week intervention.

	Ginseng				Placebo					
Sterols (nmol/mL)	Baseline	After 4 Weeks	*p* †	Change	Baseline	After 4 Weeks	*p* †	Change	*p* ‡	*p*
Cholesterol	1634.4 ± 409.4	1486.1 ± 312.8	0.002	−148.3 ± 261.1	1510.6 ± 339.2	1486.6 ± 338.0	0.543	−23.0 ± 220.5	0.039	0.047
Sitosterol	8.2 ± 8.0	7.7 ± 6.1	0.267	−0.5 ± 0.09	6.0 ± 3.2	6.1 ± 3.6	0.675	0.1 ± 1.5	0.256	0.804
Campesterol	11.3 ± 12.6	10.1 ± 9.0	0.116	−1.1 ± 4.2	8.5 ± 4.8	8.2 ± 4.6	0.256	−0.3 ± 1.6	0.311	0.937
Lanosterol	2.9 ± 1.2	3.0 ± 1.7	0.635	0.1 ± 1.6	3.0 ± 1.2	3.4 ± 3.2	0.469	0.4 ± 0.3	0.636	0.568
Desmosterol	9.6 ± 2.4	9.1 ± 2.5	0.168	−0.5 ± 2.0	10.7 ± 13.8	9.3 ± 6.3	0.328	−1.4 ± 8.0	0.501	0.551
7-DHC	6.2 ± 1.7	5.9 ± 1.8	0.137	−0.3 ± 1.1	9.4 ± 22.2	7.2 ± 9.7	0.343	−2.1 ± 12.7	0.380	0.829
7-OHC	0.3 ± 0.2	0.2 ± 0.2	0.002	−0.05 ± 0.09	0.2 ± 0.6	0.2 ± 1.4	0.908	−0.002 ± 0.1	0.047	0.063
CE14:0	14.6 ± 14.9	13.1 ± 12.8	0.393	−1.4 ± 9.9	16.9 ± 19.0	15.2 ± 17.4	0.225	−1.8 ± 8.0	0.884	0.896
CE20:4	111.9 ± 124.8	102.8 ± 107.6	0.503	−9.1 ± 80.9	99.1 ± 112.3	82.9 ± 88.1	0.238	−16.2 ± 76.2	0.711	0.459

7-DHC, 7-Dehydrocholesterol; 7-OHC, 7-Hydroxycholesterol; CE, cholesterol esters, † *p*-values were calculated using the paired *t*-test (difference after intervention within groups), ‡ *p*-values were calculated using the independent two-sample *t*-test (mean change differences between two groups). *p*-values were calculated using the analysis of covariance after adjusting for each baseline sterols.

## Supplementary Materials

The following are available online at https://www.mdpi.com/2072-6643/12/11/3423/s1, Table S1: The selective reaction monitoring transitions (Q1 and Q3 ions) and collision energies used for the sterol analyses.
