# Supplementary material for: Effect of Korean Red Ginseng on Cholesterol Metabolites in Postmenopausal Women with Hypercholesterolemia: A Pilot Randomized Controlled Trial
Source: Nutrients. 2020 Nov 8;12(11):3423. doi: 10.3390/nu12113423 (PMC7695162; doi:10.3390/nu12113423)
Supplement: Supplementary file 1 [file nutrients-12-03423-s001.pdf]

Supplementary Table S1. The selective reaction monitoring transitions (Q1 and Q3 ions) and collision energies used for the sterol analyses.

No.	Species	Adduct ion	Q1 (*m/z*)	Q3 (*m/z*)	CE (eV)	Retention time (min)
1	7-Hydroxycholesterol	$[M+H]^+$	385.4	367.3	16	2.5
2	Desmosterol	$[M+H]^+$	367.3	161.1	21	4.3
3	7-Dehydrocholesterol	$[M+H]^+$	367.3	159	25	4.5
4	Cholesterol	$[M+H]^+$	369.4	147.3	25	4.9
5	Lanosterol	$[M+H]^+$	409.4	149	29	5.3
6	Campesterol	$[M+H]^+$	383.4	147	25	5.4
7	Sitosterol	$[M+H]^+$	397.4	147.1	25	5.8
8	Cholesterol-d_7 (IS)	$[M+H]^+$	376.4	147	26	4.9
9	cholesteryl ester 14:0	$[M+Na]^+$	619.6	251.2	24	15.4
10	cholesteryl ester 20:4	$[M+Na]^+$	695.6	327.2	28	15.6
